# Exome Sequencing and Optical Genome Mapping in Molecularly Unsolved Cases of Duchenne Muscular Dystrophy: Identification of a Causative X-Chromosomal Inversion Disrupting the *DMD* Gene

**DOI:** 10.3390/ijms241914716

**Published:** 2023-09-28

**Authors:** Leoni S. Erbe, Sabine Hoffjan, Sören Janßen, Moritz Kneifel, Karsten Krause, Wanda M. Gerding, Kristina Döring, Anne-Katrin Güttsches, Andreas Roos, Elena Buena Atienza, Caspar Gross, Thomas Lücke, Hoa Huu Phuc Nguyen, Matthias Vorgerd, Cornelia Köhler

**Affiliations:** 1 Department of Human Genetics, Ruhr-University Bochum, 44801 Bochum, Germany; leoni.erbe@rub.de (L.S.E.); wanda.gerding@rub.de (W.M.G.); kristina.doering@rub.de (K.D.); huu.nguyen-r7w@rub.de (H.H.P.N.); 2 Center for Rare Diseases Ruhr (CeSER), 44791 Bochum, Germany; cornelia.koehler@kklbo.de (C.K.); thomas.luecke@rub.de (T.L.); 3 Department of Neuropediatrics, University Children’s Hospital, Ruhr-University Bochum, 44801 Bochum, Germany; soeren.janssen@rub.de; 4 Department of Neurology, Heimer Institute for Muscle Research, University Hospital Bergmannsheil, Ruhr-University Bochum, 44801 Bochum, Germany; moritz.kneifel@rub.de (M.K.); karsten.krause@ruhr-uni-bochum.de (K.K.); anne.guettsches@ruhr-uni-bochum.de (A.-K.G.); andreas.roos@uk-essen.de (A.R.); matthias.vorgerd@bergmannsheil.de (M.V.); 5 Institute of Medical Genetics and Applied Genomics, University Tübingen, 72074 Tübingen, Germany; elena.buena-atienza@med.uni-tuebingen.de (E.B.A.); caspar.gross@med.uni-tuebingen.de (C.G.); 6 NGS Competence Center Tübingen, 72076 Tübingen, Germany

**Keywords:** Duchenne muscular dystrophy, DMD, optical genome mapping, OGM, long-read sequencing, inversion, dystrophin, fukutin, FKTN, whole-exome sequencing

## Abstract

Duchenne muscular dystrophy (DMD) is a severe progressive muscle disease that mainly affects boys due to X-linked recessive inheritance. In most affected individuals, MLPA or sequencing-based techniques detect deletions, duplications, or point mutations in the dystrophin-encoding *DMD* gene. However, in a small subset of patients clinically diagnosed with DMD, the molecular cause is not identified with these routine methods. Evaluation of the 60 DMD patients in our center revealed three cases without a known genetic cause. DNA samples of these patients were analyzed using whole-exome sequencing (WES) and, if unconclusive, optical genome mapping (OGM). WES led to a diagnosis in two cases: one patient was found to carry a splice mutation in the *DMD* gene that had not been identified during previous Sanger sequencing. In the second patient, we detected two variants in the fukutin gene (*FKTN*) that were presumed to be disease-causing. In the third patient, WES was unremarkable, but OGM identified an inversion disrupting the *DMD* gene (~1.28 Mb) that was subsequently confirmed with long-read sequencing. These results highlight the importance of reanalyzing unsolved cases using WES and demonstrate that OGM is a useful method for identifying large structural variants in cases with unremarkable exome sequencing.

## 1. Introduction

Duchenne muscular dystrophy (DMD) is an X-linked neuromuscular disorder caused by lack of functional full-length dystrophin protein expression [1]. The physiological function of the dystrophin complex is to stabilize the plasma membrane of striated muscle; therefore, mutations in the *DMD* gene encoding dystrophin lead instability of the plasma membrane and myofiber loss [2]. DMD is a progressive, severe, muscle-wasting disease that presents in early childhood with proximal muscle weakness, calf hypertrophy, waddling gate, positive Gowers sign, frequent falls, and difficulties with climbing stairs or rising from the ground [3]. Despite encouraging advances in therapeutic options over the last years, life expectancy of affected individuals is still strongly reduced, with a median life expectancy of ~19 years without ventilatory support and ~29.9 years with ventilatory support [4]. The milder form of dystrophinopathy, Becker muscular dystrophy (BMD), also caused by mutations in the *DMD* gene, presents with a wider range of symptoms from limb–girdle weakness with loss of ambulation to a less severe course of disease with myalgias in youth and adulthood and is typically associated with a residual abundance of the dystrophin protein [5].

The *DMD* gene, located on the short arm of the X chromosome (Xp21.1), is composed of 79 exons and approximately 2.4 Mb in size. The vast majority of cases is caused by either exon deletions or duplications or (less frequently) point mutations in the coding regions of the *DMD* gene. For example, a single retrospective analysis of 750 Duchenne and Becker patients from southern Italy showed that 71% of DMD cases were caused by deletions of one or more exons in the dystrophin gene. Large duplications accounted for 9.73% of the mutations, and 14.93% of all mutations were point mutations [6]. For Duchenne, deletions and duplications are mostly out of frame, leading to severely reduced or absent dystrophin production, while Becker dystrophinopathy is more likely associated with in-frame deletions and duplications, granting some residual dystrophin production [7]. Likewise, as far as point mutations are concerned, the Duchenne phenotype more likely occurs with nonsense or frameshift variants, while missense or splice site variants are more likely associated with the milder Becker phenotype. However, there are deviations from these rules, and an exact genotype–phenotype correlation is not always possible, suggesting a clinical continuum of the dystrophinopathies [8].

*DMD* is one of the largest human genes, which complicated the identification of point mutations using Sanger sequencing before the era of next-generation sequencing (NGS). Nowadays, >94% of mutations are easily identified using multiplex ligation-dependent probe amplification (MLPA) and NGS-based analysis, which comprise the routine diagnostic methods for DMD and BMD. However, in approximately 2–7% of DMD cases, these standard methods still fail to identify pathogenic variants [9,10]. In some of these cases, structural variants such as insertions, balanced translocations and inversions, or deep intronic variants have been identified [6,11,12]. Additionally, differential diagnoses, including, for example, severe forms of limb–girdle muscular dystrophy (LGMD) potentially going along with secondary reduction of dystrophin in the muscle biopsy, may have to be considered in patients without mutations in the *DMD* gene, which can be identified via whole-exome sequencing (WES) [13,14].

Since mutation-specific therapeutic options for DMD (e.g., read-through therapeutics for nonsense mutations) as well as, most recently, gene therapy approaches aiming at the production of microdystrophin (https://www.fda.gov/vaccines-blood-biologics/tissue-tissue-products/elevidys, accessed on 24 June 2023) are already available or being investigated further (e.g., exon skipping methods [15,16]), unraveling the molecular pathology for each patient is highly warranted. We therefore retrospectively evaluated the data of 60 patients with a clinical diagnosis of DMD that presented in the neuromuscular clinic of the Children’s Hospital Bochum over the past 5 years and identified three cases without a known molecular cause through routine diagnostics (including at least MLPA and sequence analysis of the *DMD* gene). In these three patients, we first performed WES and secondly (if this did not lead to a diagnosis) optical genome mapping (OGM) in order to search for structural variation at the *DMD* locus. While WES can detect small variants in the coding region of genes with a high sensitivity, structural variants, especially balanced translocations or inversions, are often missed with this approach. OGM, on the other hand, is a genome-wide, amplification-free method that can detect all types of large structural variants at high resolution [17]. Together, these two methods complement each other perfectly. Two of the three cases in this study were solved via WES, revealing a previously unrecognized splice mutation in the *DMD* gene in one patient and compound heterozygous variants in the *FKTN* gene, suggesting a rare LGMD form as differential diagnosis to DMD, in the other. In the third patient, WES was inconspicuous, but OGM identified a hemizygous inversion of approximately 1.28 Mb disrupting the *DMD* gene, and immunohistochemical staining was performed to investigate the impact of this inversion on proteins involved in muscular dystrophy. These results highlight the importance of reanalyzing unsolved cases using WES and show that OGM is a useful method for identifying large structural variants in cases with unremarkable exome sequencing.

## 2. Results

### 2.1. Overview of DMD Patients in Our Center

All 60 male patients with a clinical phenotype of DMD that were treated in our center over the past 5 years had undergone routine molecular genetic testing (including at least MLPA and sequence analysis of the *DMD* gene), identifying pathogenic variants in the *DMD* gene in all but three patients. The proportion of variants within the group was as follows: 40 (~66%) patients showed a deletion of one or more exons, 3 (~5%) had a duplication of one or more exons and 14 (~23%) carried a point mutation, including 6 patients with a nonsense variant that were treated with ataluren (see Table 1). The three patients without a molecular diagnosis were further investigated in the present study.

### 2.2. Clinical Characterization of the DMD Patients without Molecular Diagnosis

#### 2.2.1. Patient 1

**Figure 1:**
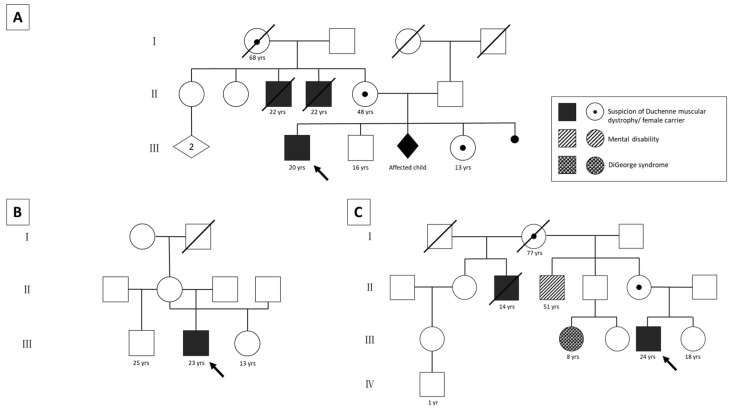
Pedigrees of the three patients. Patient 1 has a positive family history (**A**). Two maternal uncles were clinically diagnosed with DMD and died from the disease. Elevated CK levels were measured in the patient’s deceased grandmother, mother (~2000 U/L), and sister (~6400 U/L). All of them fatigue easily. One pregnancy was terminated because indirect diagnostics showed that the male fetus would most likely have been affected. The family tree of patient 2 shows an unremarkable family history (**B**). Patient 3 has a positive maternal family history (**C**). The mother’s half-brother was clinically diagnosed with DMD and died at the age of 14 years. A maternal cousin has DiGeorge syndrome (microdeletion 22q11.2), but this microdeletion was excluded in the patient’s mother. Another maternal uncle shows a mental disability that has not been investigated further. yrs: years; arrows: index patients.

Patient 1 had positive family history with two maternal uncles clinically diagnosed with DMD who were not genetically tested prior to their deaths at the age of 22 (Figure 1A). Furthermore, their maternal grandmother as well as mother and sister showed significantly elevated creatine kinase (CK) levels (sister > 6400 U/L; mother > 2000 U/L). When the mother got pregnant again, indirect testing revealed high probability that another male child would have been affected, and the pregnancy was terminated.

The patient was first found to have elevated CK levels 4 months postpartum. Significant elevations of laboratory parameters (CK > 25,000 U/L; aspartate transaminase (AST) > 590 U/L; alanine transaminase (ALT) > 550 U/L; lactate dehydrogenase (LDH) > 1900 U/L (at 1 year and 9 months)) were detected in the further course. After an initially normal development of gross motor skills, the first muscular symptoms appeared at the age of 2. The boy showed a clear deterioration in walking endurance and developed a broad-based gait pattern with frequent falls. Climbing stairs was only possible by holding on to the railing and climbing up one step at a time, which is atypical for that age. In addition, muscle pain after exertion occurred at times. The progression of muscle weakness and hypotonia, mainly in the proximal extremities, led to a loss of ambulation at 12 years of age, despite corticosteroid therapy since the age of 5.

The boy showed a positive Gowers sign at 2 years, a positive Meryon sign at 3 years, and pseudohypertrophy at 5 years of age. During the progression of the disease, contractures first developed in the upper ankle joint with pointed foot position at the age of 3, and later also in the elbow and hip joint. Additionally, cognitive and speech delay with lack of concentration became apparent. Electromyography showed evidence of myopathy.

Today, the patient is 20 years old and shows generalized, but proximally increased muscle weakness and hypotonia (Janda strength grades between 1 and 3). He is wheelchair-bound with flexion contractions in the elbow, hand, hip, knee, and upper ankle joints; however, despite of a restrictive ventilation disorder existing since the age of 18, respiratory support is not yet necessary.

**Figure 2:**
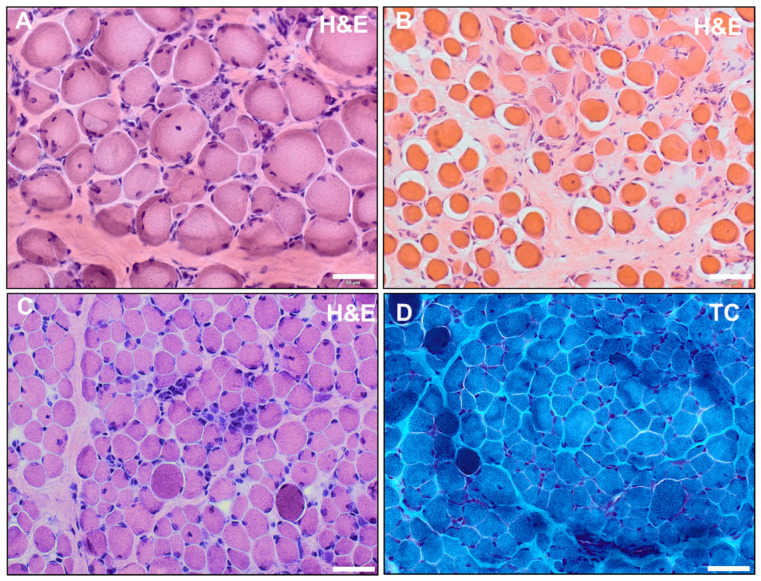
(**A**,**B**) Hematoxylin and eosin (H&E) staining of quadriceps femoris muscle of patient 1 performed at the age of 2 (**A**) and of vastus lateralis muscle of patient 2 performed at 2.5 years (**B**) showed dystrophic alterations with enhanced presence of peri- and endomysial fat cells and connective tissue (indicative of fibrosis). Moreover, fiber size variations, hypertrophic and atrophic fibers, hypercontractive fibers, internal nuclei, as well as necrotic fibers were identified. (**C**,**D**) Histochemical staining of vastus lateralis muscle of patient 3 at the age of 1 revealed dystrophic alterations with endomysial fibrosis, fiber size variations, internal nuclei, hypercontractile fibers, and small infiltrates in H&E staining (**C**); in TC staining, the hypercontractive fibers appeared as darker-stained fibers (**D**) (scalebar: 50 µm).

A muscle biopsy performed at the age of 2 showed dystrophic alterations with enhanced presence of peri- and endomysial fat cells and connective tissue (indicative of fibrosis). Moreover, fiber size variations, hypertrophic and atrophic fibers, hypercontractive fibers, internal nuclei, as well as necrotic fibers were identified (Figure 2A). The average muscle fiber diameter of type-1 fibers was 8.5 to 41 µm. For type-2 fibers, the diameter ranged from 9 to 39 µm. Immunofluorescence studies performed at the age of 2 revealed a decreased or missing expression of dystrophin using the antibodies DYS1 (against the rod domain), DYS2 (against the C-terminal region), and DYS3 (N-terminal region) but an increased expression of utrophin at the sarcolemma.

Multiplex PCR, MLPA, and Sanger sequencing of the *DMD* gene were performed at the ages of 1, 2.5, and 6 at different centers, and pathogenic variants were not detected during that time.

#### 2.2.2. Patient 2

For the second patient, the family history was unremarkable (Figure 1B). He was clinically diagnosed with DMD at the age of 1. Retrospectively, reduced intrauterine fetal movements were already evident during pregnancy.

His disease manifested at about 1 year of age by onset of muscular weakness and hypotonia of the trunk and proximal extremities. Despite some initial difficulties with crawling, he learned to walk freely at 14 months. He developed a broad-based and waddling gait and could climb stairs at first only by crawling, later, with great effort, also by holding on to the railing. He was not able to stand and jump on one leg, nor to walk on his heels. Bipedal jumping was only possible with minimal space gained between feet and floor. His blood values showed moderate elevations of laboratory parameters (CK: 3700 U/L; LDH: 1163 U/L; AST: 95 U/L; at 2.5 years). The patient developed a positive Gowers and Meryon sign and pseudohypertrophy of the calf muscles with 2.5 years. He also showed a positive Trendelenburg sign and tongue hypertrophy 3 years later. By the age of 10, independent walking was no longer possible, and he became wheelchair-bound.

The patient developed contractures in his upper ankle at almost 3 years of age, which quickly developed into pointed feet. At about 15 years of age, he was diagnosed with flexion contractures in the elbow, hip, and knee joints and at the age of 18 also in the wrist and shoulder joints. However, pronounced hypermobility of hip adduction and abduction was conspicuous. At age 15, biceps and patellar reflex were no longer triggerable, and meanwhile all muscle-intrinsic reflexes are negative. Additionally, at the age of 17, hypokinetic dysfunction of initially the right and later the left cardiac ventricle as well as a restrictive ventilatory disorder, currently not requiring therapeutic measures, were found. Furthermore, orthopedic problems such as hyperlordosis and scapula alata became apparent at the early age of approximately 3.

At present, the patient is 23 years old, and Janda strength grades of 1–2 are achieved in proximal extremities and 3–4 in distal extremities. For example, independent eating of small cut food is possible by resting elbows on the table. Intellectual development is normal; the patient has successfully obtained a qualification as an office administrator.

A muscle biopsy was performed at 2.5 years old that showed dystrophic alterations with enhanced presence of peri- and endomysial fat cells and connective tissue, internal nuclei, granular necrosis, phagocytosis, and hypercontractile fibers. Fiber size variations and fibrosis were also displayed (Figure 2B). Immunofluorescence studies revealed decreased expression of DYS1, single fibers with enhanced expression. DYS2 was partially expressed on single fibers besides negative and positive fibers. DYS3 antibody showed physiological staining.

A multiplex PCR was performed at the age of 5 and MLPA and Sanger sequencing of the *DMD* gene were performed at 15 with unremarkable results.

#### 2.2.3. Patient 3

For patient 3, the family history was positive, with a maternal uncle clinically suffering from Duchenne muscular dystrophy who died at the age of 14 (Figure 1C). Indirect genetic analysis during pregnancy revealed a high probability that the male fetus would carry the X chromosome with the causative (but unknown) *DMD* variant; however, the parents decided not to terminate the pregnancy. Additionally, a maternal cousin of the patient was diagnosed with DiGeorge syndrome, but the patient’s mother was found not to carry the familial microdeletion 22q11.2. A maternal uncle further suffered from intellectual disability of unknown cause.

Elevated CK levels were first noted at 1 month from birth, and they increased significantly during the course of the disease (up to over 17,000 U/L). First clinical symptoms such as muscle weakness and hypotonia of the proximal legs, later also of the arms, trunk, shoulders, face, and pharynx, manifested at 11 months. Later, the patient developed areflexia and multiple contractures. He showed a positive Gowers sign and pseudohypertrophy of the calves and the tongue. Motor milestones such as sitting, standing, and walking freely were achieved late. Walking without help was possible at the age of 2. The patient developed a waddling and wide-supported gait pattern and needed a wheelchair for long distances at 6 years old. In order to stem the progression of the disease, cortisone therapy was started at the age of 7. Complete loss of ambulation occurred by 11 years of age.

At 8 years and 4 months old, combined restrictive and obstructive ventilatory dysfunction was described, and from 15 years and 7 months old, CPAP ventilation was required at night and later also during the day. Since a choking event and CRP at 20 years of age, the patient is tracheotomized and permanently ventilated. A dilated cardiomyopathy was diagnosed at the age of 22. In the course of the disease, a left convex scoliosis developed as well as a hyperlordosis in the lumbar region. Decreased amplitudes of compound muscle action potentials in motoric nerve conduction studies of the tibial and median nerve were detected recently.

Currently, the patient is 24 years old. He shows severe generalized muscle weakness and hypotonia with involvement of facial and pharyngeal muscles. He is nourished through a PEG tube, tracheotomized, and ventilated the entire day. In addition to his muscular symptoms, he has a significant reduction in intelligence and dyslalia. He has never been able to complete sentences.

A muscle biopsy performed at the age of 1 showed endomysial fibrosis, fiber size variations, internal myonuclei, hypercontractile fibers, and inflammatory infiltrates (Figure 2C,D). Western blot analysis (performed at the age of 1) showed a band for dystrophin using the D1 antibody and a missing band with the DYS2 antibody. Multiplex PCR and MLPA of the *DMD* gene were performed at 6 months and 22 years old with unremarkable results.

### 2.3. Muscle Biopsy

All three patients had diagnostic muscle biopsies in early childhood (at ages 2, 2.5, and 1, respectively). For patients 1 and 2, additional material from these biopsies was not available and a repeated biopsy was rejected. For patient 3, reanalysis of the archived muscle biopsy confirmed dystrophic alterations (Figure 2C,D). Hematoxylin and eosin (H&E) staining showed endomysial fibrosis, fiber size variations, internal nuclei, hypercontractile fibers, and inflammatory infiltrates. In Gomori trichrome (TC) staining, the hypercontractive fibers appeared as darker-stained fibers. The average muscle fiber diameter was 3–45 µm (ref.: 12–25 µm).

**Figure 3:**
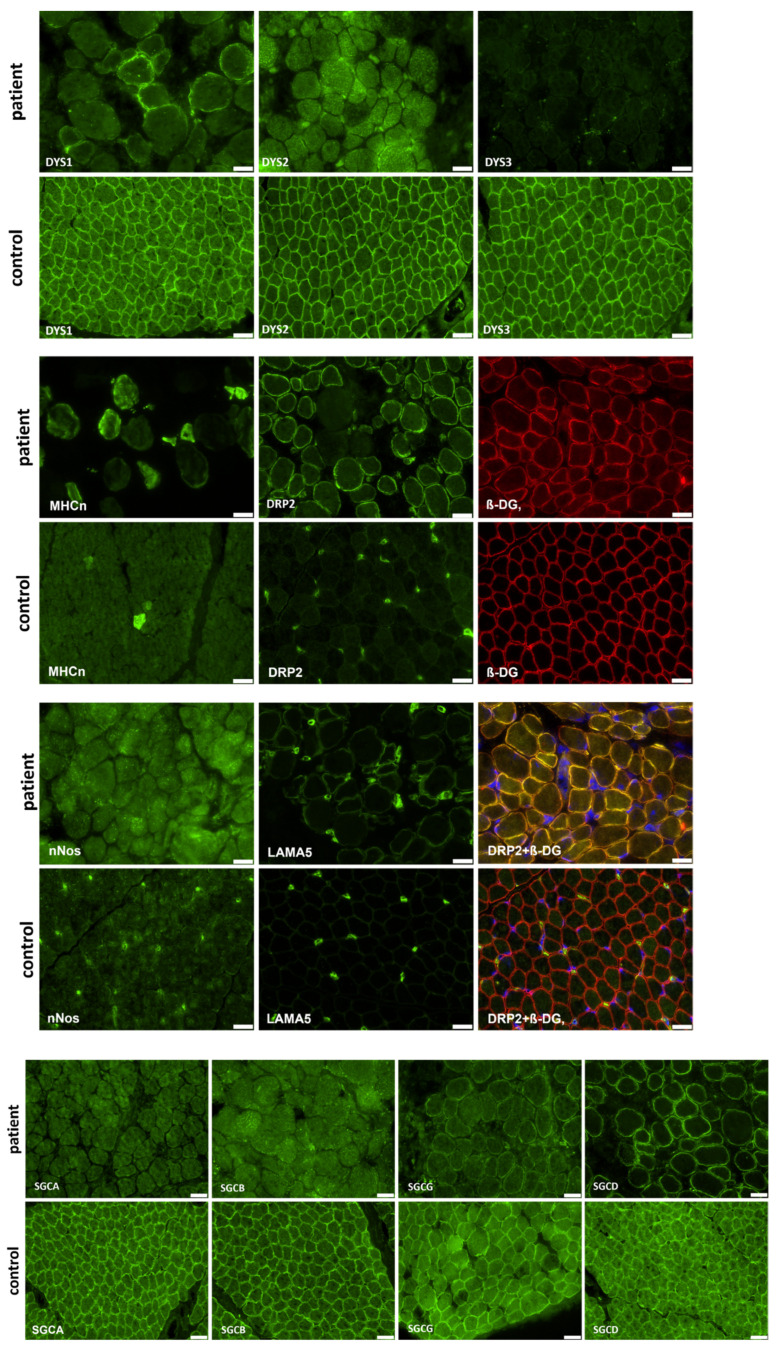
Immunofluorescence studies of vastus lateralis muscle of patient 3 showed typical alterations to proteins involved in muscular dystrophy: an absence of dystrophin (using DYS2 and DYS3 antibodies), SGCA, and SGCB, and residual staining with DYS1, SGCG, SGCD, and DRP2 antibodies. LAMA5 and nNos staining were increased. MHCn staining showed increased number of regenerative muscle fibers. DRP2 was co-stained with ß-DG and DAPI to display central nuclei (scalebar: 20 µm).

Immunofluorescence studies of vastus lateralis muscle of patient 3 showed typical alterations to proteins involved in muscular dystrophy: an absence of dystrophin using DYS2 and DYS3, as well as an absence of SGCA (α-sarcoglycan) and SGCB (β-sarcoglycan), and residual staining with DYS1, SGCG (γ-sarcoglycan), and SGCD (δ-sarcoglycan) antibodies. In comparison to an age-matched control, DRP2 (dystrophin-related protein 2) staining was increased. LAMA5 (laminin subunit alpha-5) and nNOS (nitric oxide synthase) staining were also increased. MHCn (myosin neonatal-type heavy chain) staining showed an increased number of regenerative muscle fibers (Figure 3).

### 2.4. Results of Whole-Exome Sequencing

Whole-exome sequencing identified pathogenic variants in two of the three patients. In the third patient, WES was unremarkable and OGM was performed in the next step.

The hemizygous variant c.2622+2T>G, p.? (NM_004006.3) in the *DMD* gene was detected in patient 1. This variant has not yet been described in the literature; however, it affects the highly conserved splicing region of exon 20 and was therefore classified as likely pathogenic. Sanger sequencing in the mother revealed the same variant in heterozygous state, thus confirming her carrier status for DMD, already obvious from family history. Testing of the sister was offered to the family. Why this splice variant was not identified during Sanger sequencing in the index patient performed years earlier remains unclear.

In patient 2, no pathogenic variant affecting the *DMD* gene could be identified with WES. Instead, two variants affecting the *FKTN* gene were detected. The first variant is a deletion of four nucleotides in exon 8: c.867_870del, p.(Lys290Trpfs*7) (NM_001079802.2), which has not been described in the literature yet. However, it leads to a frameshift and a premature stop codon and therefore was considered likely pathogenic. The second variant is a missense variant in exon 9 (c.920G>A, p.(Arg307Gln)) that has already been described as pathogenic several times in both homozygous and compound heterozygous states [18,19,20]. Testing the parents using Sanger sequencing revealed that the missense variant was inherited paternally, while the frameshift variant was inherited maternally, confirming the compound heterozygous state of the variants in the patient.

WES did not identify a pathogenic variant in patient 3.

### 2.5. Results of OGM and Long-Read Sequencing in Patient 3

**Figure 4:**
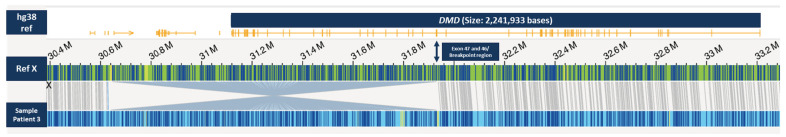
OGM Genome browser view in the *DMD* gene region on chromosome X and the location of the inversion. The green bar represents the X chromosome reference label positions (in dark blue) in the region including the *DMD* gene (with exons depicted in yellow) as shown in top line. Patient 3 (bottom line light blue bar with label positions depicted in dark blue) shows an inversion partially affecting the *DMD* gene with an estimated breakpoint region around exon 46 or exon 47.

**Figure 5:**
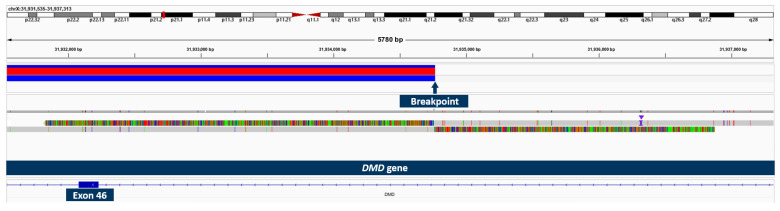
Long-read sequencing showing the breakpoint of two reads with soft-clipped sequences and structural variant called using sniffles.

Using OGM, an approximately 1.28 Mb hemizygous inversion on the X chromosome, which partially affects the *DMD* gene, was identified in patient 3 (Figure 4). The breakpoints cannot be accurately determined using OGM, as it is not a sequencing method, but the breakpoint region disrupting the *DMD* gene was displayed between exons 45 and 47. The other breakpoint appeared to be in an intragenic region. By testing the asymptomatic mother with OGM, we were able to confirm her as a heterozygous carrier of the same structural variant. Long-read sequencing specified the breakpoints (chromosome X: g.30,634,438–g.31,934,766; hg38), revealed the more precise size of 1.3 Mb, and confirmed that exons 46–79 of the *DMD* gene are affected (Figure 5). The breakpoint interrupting the *DMD* gene is located in intron 45 and the second breakpoint is found in the intergenic region between the genes *GK* and *CXorf21*. Since the inversion disrupts a large part of the C-terminal dystrophin from exon 46 to the end, we consider it pathogenic and disease-causing.

Additionally, OGM identified two other variants in patient 3 that were absent in the control dataset: a maternally inherited ~4.3 Mb duplication on chromosome 10 as well as a paternally inherited ~3 kb insertion in the *SYNE2* gene of so far unknown significance (Table 2).

## 3. Discussion

Determining the molecular cause underlying neuromuscular disorders, especially DMD, has been of increasing clinical importance over the last decade since more and more individualized and mutation-specific therapeutic approaches are already approved (e.g., ataluren for nonsense mutations in DMD) or currently under investigation [21]. In this study, we identified the genetic cause in three patients from our center that had had a clinical diagnosis of DMD for over two decades, but without molecular confirmation. In two of them, WES was able to detect the underlying molecular variation (in one actually revealing a different diagnosis), while the third case was solved with OGM and subsequent long-read sequencing.

In patient 1, a so far unrecognized splice variant in the *DMD* gene was identified. It remains unclear why this variant was not detected during Sanger sequencing performed years earlier. However, this case demonstrates that for patients with negative Sanger sequencing results dating back many years, reevaluation with WES methods seems a reasonable option.

Patient 2 was clinically diagnosed with DMD in childhood, which could not be confirmed genetically. However, retrospectively, one could argue that, in this patient, the muscle biopsy was not completely conclusive, with physiological staining of DYS3 and single fibers of enhanced expression of DYS1, rather suggesting Becker muscular dystrophy, which symptomatically, on the other hand, was not compatible with the phenotype of the patient. Also, CK levels were not as high as in the two other patients, and the family history was unremarkable (which does not contradict the clinical diagnosis DMD, since approximately 30% occur as de novo mutations [1]). WES performed in this study finally revealed two variants in the *FKTN* gene that are most likely causative for the disease. Pathogenic *FKTN* variants are associated with different phenotypes, for example, the severe Fukuyama congenital muscular dystrophy (FCMD) [22]. Since our patient does not have any intellectual disability, his phenotype is more consistent with a milder limb–girdle form of clinical manifestation. Of note, there are already some patients described with compound heterozygous or homozygous *FKTN* variants and a limb–girdle dystrophy–dystroglycanopathy [23]. The p.(Arg307Gln) variant, which was identified in our patient, has already been described in homozygous state in a patient who presented with loss of ambulation at the age of 11 [19]. Two siblings carrying a duplication in addition to the p.(Arg307Gln) variant were also described as responding very well to steroid therapy [18]. Our patient’s phenotype, which is associated with an inability to walk, may be due to the combination with the frameshift variant on the second allele.

Despite a clear clinical diagnosis and histopathology, MLPA, Sanger sequencing of the *DMD* gene, and WES did not identify a pathogenic variant in patient 3. To screen the genome for large structural variants (SVs) that could not be detected using these methods, we analyzed the patient’s DNA with optical genome mapping, a novel method in which high molecular weight (HMW) DNA is linearized in nanochannels via electrophoresis and is imaged using florescence microscopy. Using OGM, the third patient was found to carry an X-chromosomal inversion disrupting the *DMD* gene that has not been previously reported. Large-scale inversions seem to be a very rare but already identified cause of Duchenne muscular dystrophy: most recently, five inversions affecting the *DMD* gene were detected using NGS including long-read whole-genome sequencing [24,25,26].

Optical genome mapping has proven a useful addition in genetic diagnostics. It has been demonstrated that structural variants including inversions are reliably found with OGM [27]. One advantage of OGM is that all types of structural variants can be detected at high resolution. Insertions and deletions can be detected from 500 bp [28], inversions and duplications from 30 kb, and translocations from 50 kb. In comparison, the resolution of classical karyotyping is only about 4–5 Mb. Microarrays offer a better resolution but are not suitable for detecting complex or balanced structural variants such as inversions [29]. Another advantage is that optical genome mapping analyzes native DNA, thus eliminating amplification bias. In AML/MDS patients, OGM showed a clear benefit compared to classical cytogenetic methods such as karyotyping, FISH, and microarrays by detecting additional variants [30]. Success has also been achieved in the diagnostics of hereditary diseases. Recently, 100% concordance was observed in the evaluation of 99 chromosome aberrations with non-centromeric breakpoints in constitutional diseases compared to standard diagnostics [31]. Genetic causes were identified in cases with clear clinical evidence for a monogenic disease in which routine diagnostics remained unremarkable, e.g., a translocation concerning the *FBN1* gene in Marfan syndrome, a mosaic deletion of the *CDKL5* gene associated with developmental and epileptic encephalopathy 2, and an inversion of the *NF1* gene in neurofibromatosis type 1 [32,33,34]. Furthermore, there is great potential for OGM in the field of prenatal diagnostics, as it is possible to detect different structural variants including fragile X expansion with a single assay [35]. Especially in the detection of large structural variants that are difficult to analyze using long-read sequencing and are too small for karyotyping, OGM is a diagnostic enrichment. Currently, the method is limited by the fact that it is not yet a high-throughput technique, and it takes about 48 h for a sample to be ready for analysis. Further, some areas, such as the centromere, telomere, and regions with low label density, cannot be adequately analyzed with OGM at this time [36].

While analyzing the data with a focus on neuromuscular genes, we found two other variants in our patient that were absent in the control dataset: a maternally inherited ~4.3 Mb duplication on chromosome 10 as well as a paternally inherited ~3 kb insertion in the *SYNE2* gene of so far unknown significance. The region of the ~4.3 Mb duplication comprises numerous genes, including the genes *CHAT* and *SLC18A3* that are associated with presynaptic congenital myasthenic syndrome 6 and 21, respectively [37,38], and the *SYNE2* gene, which is associated with Emery–Dreifuss muscular dystrophy 5 [39]. Based on the detection in the parental genome and the mismatched phenotype, we prefer not to assume pathological relevance of these variants. However, it cannot be fully excluded that the large duplication on chromosome 10 could somehow be connected to the rather severe intellectual disability in patient 3, which appears more profound than for most DMD patients. While most Duchenne patients have no mental impairment, it is observed in about one third [40]. The inversion disrupts the distal part of the gene with exon 46–79. Thus, all isoforms of the *DMD* gene are affected by the inversion, including Dp140 and Dp71, which are present in the brain. The distal region after exon 63 is associated with the most severe mental impairment, as mutations here lead to a loss of Dp71 [41,42]. We therefore suggest that the C-terminal localization of the structural variant leads to a loss of Dp71 that accounts for the severe cognitive impairment in our patient. However, additional factors cannot be excluded.

The immunofluorescence studies showed residual staining of the rod domain and an absence of the N-terminal region of dystrophin although these proteins were not affected by the inversion of the *DMD* gene (Supplementary Figure S1). This histological finding is contradictory to the assumption that the identified inversion should lead to a decrease in/loss of immunoreactivity for DYS2 corresponding to the last 17 amino acids of the DMD protein, which are affected by the genetic alteration. One might assume that this proteinogenic finding arises from the utilization of different promotors toward the expression of shortened DMD isoforms. However, further transcript studies on muscle RNA are needed to confirm this assumption. Nevertheless, the concomitant dysregulation of proteins belonging to the dystroglycan complex (such as sarcoglycans) support the concept of a pathogenicity of the identified inversion and along this line suggest a vulnerability in the overall dystroglycan complex, a known myopathological hallmark in DMD.

In comparison to an age-matched control, DRP2 staining was increased in our DMD patient. The family of dystrophin-related proteins (DRPs) consist of DRP (utrophin), DRP2, and dystrobrevin. All these proteins share functional motifs and sequence similarities with dystrophin. DRP2 is encoded in humans by a 45 kb gene localized to Xq22. Its overall structure is similar to the Dp116 dystrophin isoform [43]. Thus, the upregulation of the DRP2 protein could be a compensating mechanism to rescue dystrophin-deficient muscle, as DRP does [44].

In conclusion, we were able to find the molecular cause in two of three previously unsolved DMD cases with WES, one of them leading to a different diagnosis, and the third one with OGM. These results highlight the importance of reanalyzing unsolved cases using WES and show that OGM is a useful method for identifying large structural variants in cases with unremarkable exome sequencing.

## 4. Materials and Methods

### 4.1. Patients

Sixty male patients with a clinical phenotype of DMD (defined as proximal leg progressive muscle disease with onset between infancy and primary school age, loss of ambulation in adolescence, lung and heart involvement, elevated laboratory values (CK elevated at least 10-fold), and absent or severely reduced dystrophin in muscle biopsy) were treated at the neuromuscular clinic of the Children’s Hospital in Bochum and/or the University Hospital Bergmannsheil over the last 5 years. Medical records were reviewed retrospectively for data assessment. All patients had undergone routine molecular genetic testing (including at least MLPA and sequence analysis of the *DMD* gene). The three patients with suspected DMD but no molecular diagnosis were further investigated, including a detailed clinical history, clinical examination and retrospective evaluation of blood tests, electroneurography, electromyography, MRI, and muscle biopsy. All patients/families provided informed consent for this study and the publication of its results.

### 4.2. Muscle Biopsy

Only for patient 3 was material from the muscle biopsy at the age of 1 still available for reevaluation. H&E staining was performed following standard procedures [45]. First, unfixed serial skeletal muscle cryosections of 10 µm thickness were incubated with hematoxylin for 30 s, followed by washing steps and dehydration steps with ethanol. Then, sections were incubated with eosin for 10 s and washed in several steps with ethanol and xylol.

Immunofluorescence studies were performed on muscle samples of patient 3 collected at one year of age. Frozen skeletal muscle sections were incubated overnight at 4 °C with primary antibodies against 13 proteins involved in muscular dystrophy (Table 3) followed by several washing steps and incubation with isotype-specific secondary antibodies conjugated with Alexa Fluor 488-conjugated goat anti-mouse IgG (#115-545-146), (dilution = 1:250) and goat anti-rabbit IgG (#111-545-144), (dilution = 1:500) or Alexa 594-conjugated goat anti-mouse IgG (#115-585-068, dilution = 1:500). Nuclei were visualized using 4′,6-diamidino-2-phenylindole staining.

Images were recorded using an Olympus microscope (Olympus IX83, Olympus, Hamburg, Germany) with connected cameras (XM10 for immunofluorescence and XC50 for H&E; Olympus, Tokyo, Japan) and CellSens software (CellSens Dimension version 1.17) for data processing.

### 4.3. Whole-Exome Sequencing and Segregation Analyses

Whole-exome sequencing (WES) libraries were constructed using the Twist Comprehensive Exome/Twist Library Preparation Kit (V2) (Twist Bioscience, San Francisco, CA, USA) and sequenced as 2 × 150 nt paired end reads on a NextSeq1000 instrument (Illumina, San Diego, CA, USA). Sequence alignment, variant calling, and annotation were performed using the Varvis bioinformatics pipeline v1.22 (Limbus Medical Technologies GmbH, Rostock, Germany). Detected variants were evaluated based on the ACMG classification system [46] and the patient’s phenotype. Segregation analyses were performed with Sanger sequencing (information on primers available upon request).

### 4.4. Optical Genome Mapping

Whole blood was drawn into an EDTA tube and frozen at −40 °C within 66 h. High molecular weight (HMW) DNA was isolated from the frozen EDTA blood using the Bionano SP Blood and Cell Culture DNA Isolation Kit v2 according to manufacturer’s instructions (Bionano Genomics, San Diego, CA, USA). Specific sequences of the DNA sample were labeled using the Direct Label and Stain kit, which includes the direct labeling enzyme DLE-1 and the DL-green fluorophores. The labeled HMW DNA was loaded on a Bionano Genomics Saphyr Chip G2.3 for linearization and imaging using a Saphyr instrument (Bionano Genomics, San Diego, CA, USA). DNA was linearized in nanochannels via electrophoresis and imaged using fluorescence microscopy.

Bioinformatic processing and analysis was performed using Bionano Solve 3.7.2 and Bionano Access 1.7.2 software. All molecular quality report (MQR) values, including a minimum effective coverage of 80×, were within range according to the manufacturer’s instructions. The de novo assembly pipeline was used to detect hereditary and de novo variants affecting one or two alleles in the patient’s genome. During the analysis, labeling patterns between the constructed patient’s genome map and a GRCh38/hg38 reference genome map were compared. We filtered the identified SVs using the default filter settings. We examined structural variants that were present in 1% or less of the control datasets in the manufacturer’s control population in terms of whether they were related to the patient’s phenotype. The variants reported here affect genes related to neuromuscular diseases or phenotypes (using a BED file containing 493 genes associated with neuromuscular proteins and diseases). The control population includes de novo assembly DLE-1 datasets from 179 ethnically diverse individuals.

### 4.5. Long-Read Sequencing

Genomic integrity was assessed using pulse-field capillary electrophoresis with the Genomic DNA 165 kb Analysis Kit on a FemtoPulse (Agilent, Santa Clara, CA, USA) instrument. Quantitation of DNA was assessed using the dsDNA High Sensitivity assay on a Qubit 3 fluorometer (Thermo Fisher, Waltham, MA, USA). A total of 2.4 µg of genomic DNA was used for preparing the library with the 1D Ligation SQK-LSK109-XL Sequencing kit (Oxford Nanopore Technologies, Oxford, UK). A total of 40 fmol library was loaded on a single PromethION R9 flow cell. A nuclease flush was performed to reload the library. Reads were mapped with minimap2 (v2.2.4, default parameters) to the reference genome GRCh38 and structural variants called with sniffles2 (v2.0.2) [47,48].

## Figures and Tables

**Table 1:** Type of variants in the DMD gene of 60 patients with a clinical Duchenne phenotype treated in the neuromuscular clinic of the Children’s Hospital Bochum over the past 5 years.

Type of Variants in the *DMD* Gene	Count (%)
Deletion of one or more exons	40 (~66%)
Duplication of one or more exons	3 (~5%)
Point mutation Nonsense mutation	14 (~23%) 6 (~10%)
No mutation detected	3 (~5%)
Total	60

**Table 2:** Structural variants (SVs) and copy number variants (CNVs) found in muscle genes in the genome of patient 3 using OGM.

Chromosome	Size and Type	Start Labeling Position (hg38)	End Labeling Position (hg38)	Muscle Genes	Parents
X	~1.28 Mb Inversion	30646419	31930149	*DMD*	Mother as heterozygous carrier
10	~4.3 Mb Duplication	45702207	50040591	*CHAT, SLC18A3*	Maternally inherited
14	~3 kb Insertion	63964082	63991908	*SYNE2*	Paternally inherited

**Table 3:** Antibodies used in immunohistochemical staining.

Antibody	Dilution	Abbreviation	Supplier
DYS1, mmc	1:3	NCL-DYS1, Lot: 6066097	Novocastra
DYS2, mmc	1:10	NCL-DYS2, Lot: 6065996	Novocastra
DYS3, mmc	1:10	NCL-DYS3, Lot: 6066797	Novocastra
Alpha-sarcoglycan, mmc	1:50	NCL-L-a-SARC,	Novocastra
Beta-sarcoglycan, mmc	1:50	NCL-L-b-SARC, Lot: 6083740	Novocastra
Gamma-sarcoglycan, mmc	1:25	NCL-g-SARC, Lot: 6084768	Novocastra
Delta-sarcoglycan, mmc	1:10	NCL-d-SARC, Lot: 6069438	Novocastra
LAMA5, mmc	1:500	MAB1924 Lot: 21031281	Chemicon, Merck
DRP2, mmc	1:5	NCL-DRP2, Lot: 6035452	Novocastra
MHCn, mmc	1:5	NCL-MHCn, Lot: 6091144	Novocastra
Beta-dystroglycan, mmc	1:10	NCL-b-DG	Novocastra
nNos, rpc	1:200	06-528, Lot: #18537	Upstate biotechnology
Spectrin, mmc	1:100	NCL-SPEC1, Lot: 6084548	Novocastra

(mmc: mouse monoclonal; rpc: rabbit polyclonal).

## Data Availability

The data presented in this study are available upon request from the corresponding author.

## Supplementary Materials

The following supporting information can be downloaded at: https://www.mdpi.com/article/10.3390/ijms241914716/s1.
