# Supplementary material for: Exome Sequencing and Optical Genome Mapping in Molecularly Unsolved Cases of Duchenne Muscular Dystrophy: Identification of a Causative X-Chromosomal Inversion Disrupting the DMD Gene
Source: Int J Mol Sci. 2023 Sep 28;24(19):14716. doi: 10.3390/ijms241914716 (PMC10572545; doi:10.3390/ijms241914716)
Supplement: Supplementary file 1 [file ijms-24-14716-s001.zip › ijms-2616470-supplementary.pdf]

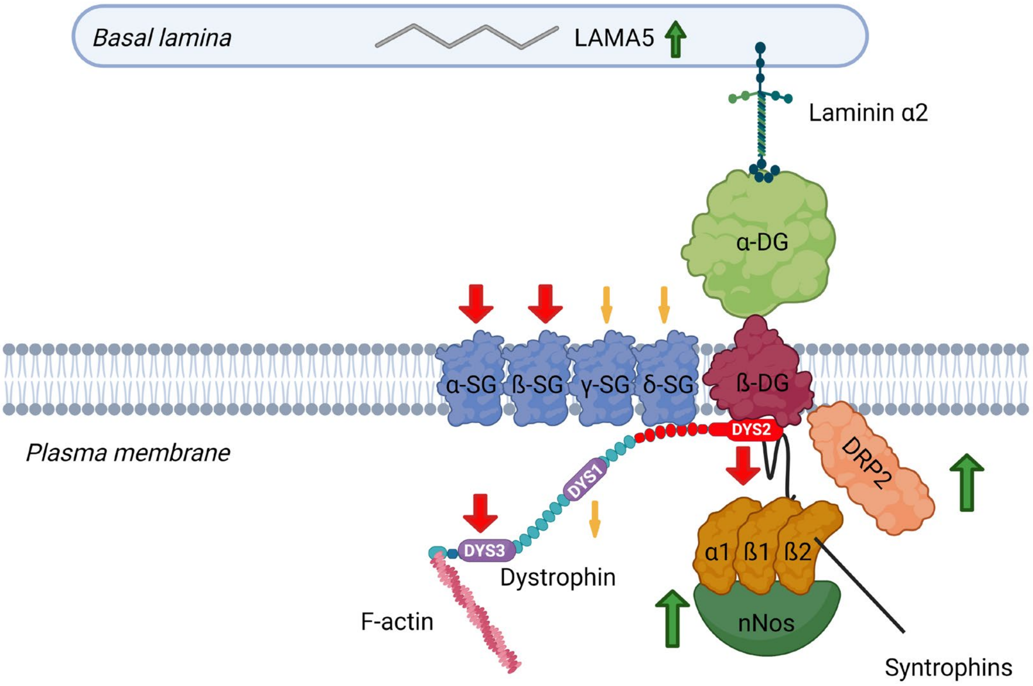

Supplementary Figure S1. Schematic figure of the dystrophin sarcoglycan complex: absence of proteins in immunofluorescence studies (red arrows), residual staining of proteins (yellow arrows), increased staining of proteins (green arrows) in patient 3. Red part of the dystrophin: schematic location of the inversion of patient 3 (created with BioRender.com).
